# The archaeal “7 kDa DNA-binding” proteins: extended characterization of an old gifted family

**DOI:** 10.1038/srep37274

**Published:** 2016-11-17

**Authors:** Valentina Kalichuk, Ghislaine Béhar, Axelle Renodon-Cornière, Georgi Danovski, Gonzalo Obal, Jacques Barbet, Barbara Mouratou, Frédéric Pecorari

**Affiliations:** 1 CRCNA, Inserm, CNRS, Université d’Angers, Université de Nantes, Nantes, France; 2 Université catholique de Louvain, Louvain Drug Research Institute, Advanced Drug Delivery and Biomaterials, Brussels, Belgium; 3 Institut Pasteur de Montevideo, Protein Biophysics Unit, Montevideo, Uruguay

## Abstract

The “7 kDa DNA-binding” family, also known as the Sul7d family, is composed of chromatin proteins from the *Sulfolobales* archaeal order. Among them, Sac7d and Sso7d have been the focus of several studies with some characterization of their properties. Here, we studied eleven other proteins alongside Sac7d and Sso7d under the same conditions. The dissociation constants of the purified proteins for binding to double-stranded DNA (dsDNA) were determined in phosphate-buffered saline at 25 °C and were in the range from 11 μM to 22 μM with a preference for G/C rich sequences. In accordance with the extremophilic origin of their hosts, the proteins were found highly stable from pH 0 to pH 12 and at temperatures from 85.5 °C to 100 °C. Thus, these results validate eight putative “7 kDa DNA-binding” family proteins and show that they behave similarly regarding both their function and their stability among various genera and species. As Sac7d and Sso7d have found numerous uses as molecular biology reagents and artificial affinity proteins, this study also sheds light on even more attractive proteins that will facilitate engineering of novel highly robust reagents.

In living organisms, the long genomic DNA has to be packed in order to fit into cells, while the genetic information must stay accessible for replication and transcription events. To this aim, organisms have developed different compaction systems, such as the wrapping of DNA around histones to form the chromatin in Eukarya, and the supercoiling of DNA with the help of non-histone proteins to form the nucleoid in Bacteria. Archaea often live in extreme environments and have the additional challenge to protect their genomic DNA from extreme conditions, such as high temperatures.

Many Archaea contain homologs of eukaryotic histones, but *Desulfurococcales*, *Thermoplasmatales* and *Sulfolobales* use a different kind of packaging proteins^1^,^2^. Hyperthermophile and acidophile archaea of the *Sulfolobales* order from the *Crenarchaeota* kingdom express small basic DNA-binding proteins, which represent about 5% of the total soluble cellular proteins, sufficient to coat the entire genome of a *Sulfolobus* cell^3^. These proteins constitute the family called “7 kDa DNA-binding” or Sul7d^4^. They were first isolated from *Sulfolobus acidocaldarius* which produces five of them, named Sac7a, b, c, d, and e. Sac7d and Sac7e are encoded by distinct genes, while Sac7a and b are truncated versions of Sac7d^5^,^6^,^7^. Highly similar homologs have been found in all *Sulfolobus* species, such as Sso7d from *Sulfolobus solfataricus*^8^, and Ssh7a and Ssh7b from *Sulfolobus shibatae* - two proteins encoded by two distinct genes^3^,^9^. Sac7d and Sso7d are the two most studied proteins of this family. They have been characterized for their structure, function, chemical stability and biophysical properties^7^. Sac7d and Sso7d are hyperthermostable (*T*_m_ = 90.4 °C and 100.2 °C, respectively)^10^,^11^ and are resistant from pH 0 up to at least pH 12^12^,^13^. Although Sac7d and Sso7d sequences show only few differences, Sso7d is more stable than Sac7d. Their three-dimensional structures show that they both fold as an SH3-like domain capped by a C-terminal α-helix^14^,^15^ and that they sharply kink the double DNA helix upon binding into the minor groove^16^,^17^. It has been shown that Sac7d and Sso7d are general dsDNA binders with *K*_*D*_ values varying in a salt dependent manner from 20 nM (low salt) to 3.8 μM (high salt) for Sac7d, and from 116 nM to 12.8 μM for Sso7d, and with a preference for G/C rich sequences^18^,^19^. Sac7d has the property to increase the thermal stability of DNA duplexes by as much as 43.5 °C^6^,^15^. Furthermore, Ssh7a and Ssh7b have been partially characterized and an affinity for dsDNA of about 100 nM was reported in a low salt buffer^9^. Thus, the biological role of these chromatin proteins is most probably to bind genomic DNA in order to prevent its melting at the high growth temperatures of these Archaea (~75–85 °C)^6^.

The combined high stability and ability to bind any dsDNA sequences of the “7 kDa DNA-binding” proteins have paved the way for developing improved tools for molecular biology by the genetic fusion of the genes encoding Sso7d and various enzymes, some of them being now marketed^20^. Furthermore, proteins from the “7 kDa DNA-binding” family have been described by our group as the first archaeal scaffolds used for the generation of artificial affinity proteins^21^,^22^, that we named Affitins^23^. Two other research groups confirmed independently the interest of this approach^24^,^25^.

Although the “7 kDa DNA-binding” proteins are important for the biology of *Sulfolobales* and for various applications, this family remains poorly characterized with putative proteins from genera such as *Metallosphaera* and *Acidianus*. Whether these members have similar DNA binding properties and robustness remains an open question. Here, in addition to Sac7d and Sso7d, we report the production and characterization of a set of eleven sequences from this family of proteins under the same *in vitro* conditions to allow comparison of their properties. We have determined their dissociation constants for DNA as well as their thermal and pH stabilities. This study reveals interesting common properties as well as differences and gives new insights about this family of proteins.

## Results

### Choice of protein set and sequence alignment

**Figure 1:**
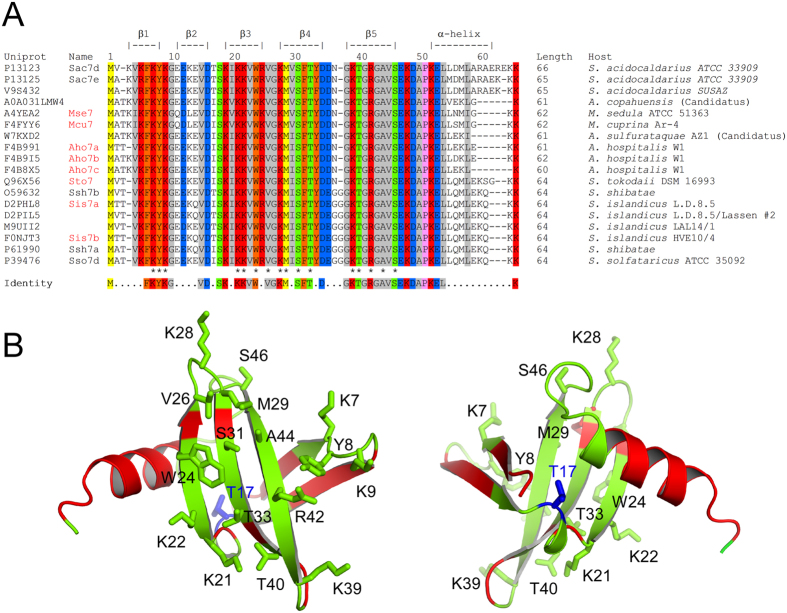
Sequence analysis of Sac7d homologs. (**A**) The multiple-sequence alignment was performed by T-Coffee^50^ and was colored using “The Sequence Manipulation Suite”^51^ (grey: G, A, V, L, I; orange: F, Y, W; yellow: M; green: S, T; red: K, R, H; blue: D, E; brown: N, Q; pink: P) to highlight residues which are identical or similar, according to their biochemical properties, for all sequences at a given position. The residues involved in the dsDNA binding according to the three-dimensional structure of Sac7d are indicated with an asterisk^16^. Amino-acid numbering is according that of Sac7d. For each homologous protein are indicated: the Uniprot accession number, the common name when known in black (and the names we proposed in red for the proteins that were not characterized before this study), the sequence length and the hosting archaea. The sequences of the proteins studied in this work have a region “RGSHHHHHHGS” inserted just after the initial methionine and “LN” after the last lysine residue resulting from their sub-cloning and allowing their purification and detection. (**B**) Two orientations of the three-dimensional structure of Sac7d (pdb code 1AZP) representing identical residues among the thirteen proteins (green), those which are not conserved (red) and Thr17 (blue). The side chains of residues which are involved in DNA interaction are depicted as green sticks.

The *Sulfolobales* order is comprised of the genera *Sulfolobus*, *Acidianus*, *Metallosphaera*, *Stygiolobus*, *Sulfurisphaera*, and the proposed novel *Candidatus Aramenus* genus that has been recently reported^26^. A search for protein sequences homologous to Sac7d in the Uniprot database returned 48 sequences from Archaea, 18 being unique and belonging to the *Sulfolobus*, *Acidianus*, *Metallosphaera,* and the proposed *Candidatus Aramenus* genera. The analysis of the multiple sequences alignment for these 18 proteins showed that their sequences mostly differ at their N- and C-terminus and share 71 to 98% identity according to the “Sequence Identity And Similarity” tool available online (http://imed.med.ucm.es/Tools/sias.html). For further analysis, the amino-acids were divided into eight standard similarity groups based on their common characteristics, defined by the side chains: GAVLI (aliphatic without proline), FYW (aromatic), CM (sulphur-containing), ST (hydroxylic), KRH (basic), DE (acidic) NQ (amidic), P (proline). By analysing the distribution of these eight groups among the sequences, the proteins showed a similarity comprised between 83% and 100% (Fig. 1A). Interestingly, all amino-acids known to interact with dsDNA are conserved (Fig. 1A). In addition to Sac7d and Sso7d, eleven representative members of the “7 kDa DNA-binding” family, including eight putative ones, were chosen for this study. We propose to name those from *Acidianus hospitalis* as Aho7a, Aho7b, Aho7c, and those from *Sulfolobus islandicus* as Sis7a, and Sis7b, as the proteins from this family are traditionally named according to the organism from which they were isolated followed by a lowercase letter in order of increasing basicity if several copies exist in cells (Fig. 1). The proteins from *Metallosphaera sedula* and *Metallosphaera cuprina* were named Mse7 and Mcu7 as we found only one copy of a *sul7* gene in their respective genomes. In the genome of *Sulfolobus tokodaii,* we found two copies of a *sul7* gene with a difference of only one base, encoding the same protein that we named Sto7.

While the three β-sheets most involved in the DNA-binding interaction are perfectly conserved (Fig. 1B), two main variations can be seen among the studied proteins: i) a longer loop between β-sheets 4 and 5, with three consecutive glycines for Ssh7b, Sis7a, Sis7b, Ssh7a and Sso7d; and ii) the length of the α-helix, being twice shorter in the smaller homologs compared to Sac7d. Some particular sequences can be highlighted here: Sto7, which has a quite similar α-helix to those of Ssh7b, Sis7a, Sis7b, Ssh7a, and Sso7d without having the “GGG loop”, Aho7c which is the only protein that does not have the otherwise conserved penultimate lysine; and finally Mse7, Mcu7, Aho7a, Aho7b, and Aho7c which have shorter helices.

All studied proteins possess sequence lengths ranging from 60 to 66 amino-acids (*i.e.* the minimal and maximal lengths known for this family) and they originate from three archaeal genera known to host proteins from this family, *Sulfolobus*, *Metallosphaera*, and *Acidianus.* Three sequences available for *Acidianus hospitalis* were studied as they correspond to several genes of the same W1 strain. Furthermore, being the shortest members of the “7 kDa DNA-binding” family, these three proteins are particularly attractive considering the importance of a minimal size for biomedical applications. The proteins from *Candidatus Acidianus copahuensis*^27^ and from *Candidatus Aramenus sulfurataquae*^26^ were not studied as their sequences were released after the beginning of this study.

### Production of soluble proteins

The proteins were produced in the cytoplasm of *Escherichia coli (E. coli*) with an expression induced for 3 h at 30 °C. Generally it is possible to purify the proteins of this family without affinity tags due to their expected high temperature and acidic stabilities^7^. However we chose to use N-terminal His6-tagged variants in order to facilitate their detection, for instance in enzyme-linked immunosorbent assays (ELISA). The proteins could be purified to homogeneity by immobilized metal ion affinity chromatography (IMAC), followed by gel filtration. They showed the expected molecular weight (~9 kDa) according to sodium dodecyl sulfate-polyacrylamide gel electrophoresis (SDS-PAGE) (see Supplementary Fig. S1A) in line with molecular weights calculated from their sequences. The molecular weights of proteins were confirmed by mass spectrometry analysis (Table 1). Furthermore, all proteins were eluted as a sharp symmetric peak from the size-exclusion chromatography at the volume corresponding to monomeric species of about 9 kDa (see Supplementary Fig. S1B). This result suggests that the proteins had a globular conformation compatible with a native state. Production yields were in the range of 5–10 mg of purified protein per liter of culture (Table 1).

### DNA binding properties

#### dsDNA binding activity

**Figure 2:**
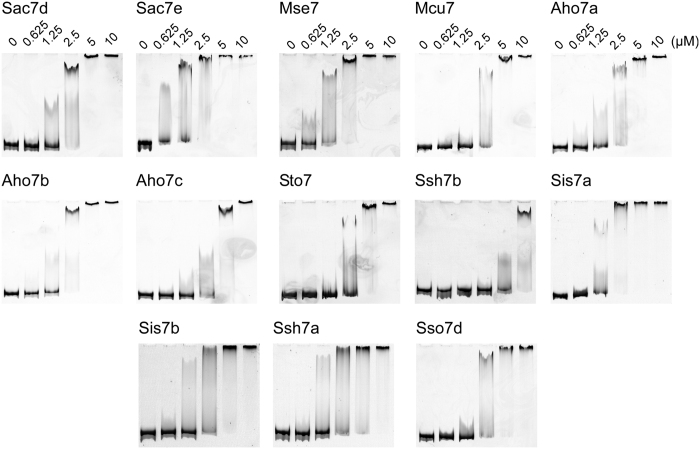
DNA-binding properties of proteins. Electrophoretic mobility shift assay (EMSA) of the proteins at 0, 0.625, 1.25, 2.5, 5 and 10 μM following incubation with ct-DNA. An electrophoretic mobility shift of the dsDNA was observed in presence of proteins at concentration higher or equal to 5 μM.

The DNA-binding properties of the proteins were first studied by electrophoretic mobility shift assay under non-denaturing conditions. In these experiments, a fixed concentration of a 415 bp PCR product was incubated with various concentrations of the proteins and the resulting complexes were analyzed on polyacrylamide gels stained with Gel-Red (Fig. 2). As expected for the “7 kDa DNA-binding” family, all proteins bound to dsDNA, and a reduced mobility of the dsDNA band was observed for protein concentrations ≥0.625 μM (Sac7e, Mse7), ≥1.25 μM (Sac7d, Aho7a, Aho7b, Aho7c, Sis7a, Sis7b, Sso7d, Ssh7a), ≥2.5 μM (Mcu7, Sto7) and ≥5 μM (Ssh7b). These results confirmed that all expressed recombinant proteins were functional.

#### Determination of affinity for dsDNA

The binding of proteins to double-stranded DNA from calf-thymus (ct-DNA) was followed at 25 °C by monitoring the change in fluorescence quenching of their single tryptophan (see Supplementary Fig. S2). The affinities were determined using a mathematical model developed for non-cooperative binding of proteins to DNA that was used in previous studies^18^. All proteins were shown to bind ct-DNA. The dissociation constants were quite similar and in the range from 11 to 22 μM (Table 1), with an average value of 16 μM. Ssh7b had the lowest affinity for ct-DNA (*K*_*D*_ = 22 μM) and Sac7e the highest affinity (*K*_*D*_ = 11 μM). The binding site sizes (n) were about 6–8 bases per protein (Table 1), as previously reported for Sac7d and Sso7d^18^,^19^.

#### Sequence selectivity of proteins for dsDNA

**Figure 3:**
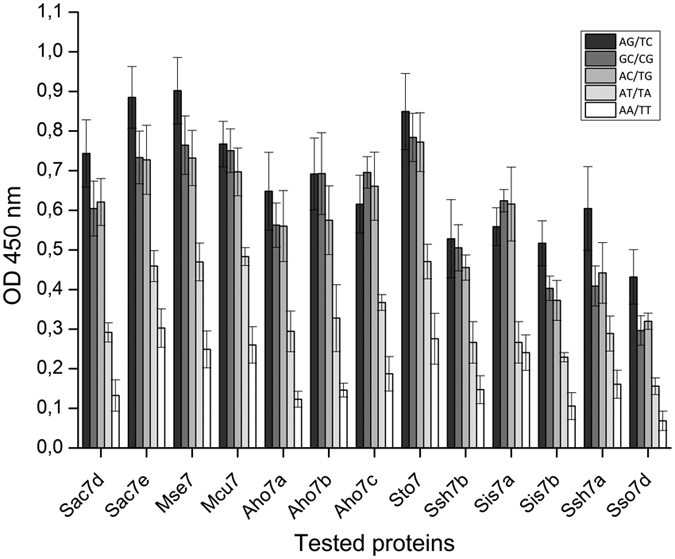
Sequence selectivity of proteins for dsDNA. Proteins show similar binding preferences by ELISA. Plates were coated with 1 μg/mL Neutravidin and 2.5 ng per well of biotinylated dsDNA were immobilized. Proteins were added at 200 nM. Binding of proteins to dsDNA was detected with anti-RGS(His)6-HRP antibody conjugate. As the recorded absorbance is proportional to the amount of bound protein, higher values correspond to higher affinities for dsDNA. Results are representative of 3 experiments.

Enzyme-linked immunosorbent assay (ELISA) for dsDNA sequence selectivity was designed by preparing several dsDNA: [poly(dAdT)]_2_, poly(dAdC).poly(dGdT), poly(dAdG).poly(dCdT), [poly(dGdC)]_2_, and poly(dA).poly(dT). Collectively, the dsDNA thus obtained contained various and significantly different sequences, corresponding to 18 different triplets and quadruplets, to test specificity of the proteins. The assays were first performed using phosphate buffered saline, pH 7.4 (PBS), but the micromolar affinity prevented the observation of a signal. As it is known for Sac7d and Sso7d that their affinities for dsDNA are in the nanomolar range in low ionic strength buffers^15^,^18^, we performed ELISA in 10 mM KH_2_PO_4_, 50 mM KCl, pH 7. A dissociation constant of 203 nM was determined for Sac7d with ct-DNA in this buffer, a value compatible with ELISA assays (see Supplementary Fig. S3). Figure 3 shows the results of protein addition to dsDNA products after analysis by ELISA. All proteins showed binding to each dsDNA sequences with a preference for those being G/C rich.

### Biophysical properties of the proteins

#### Circular dichroism spectra

**Figure 4:**
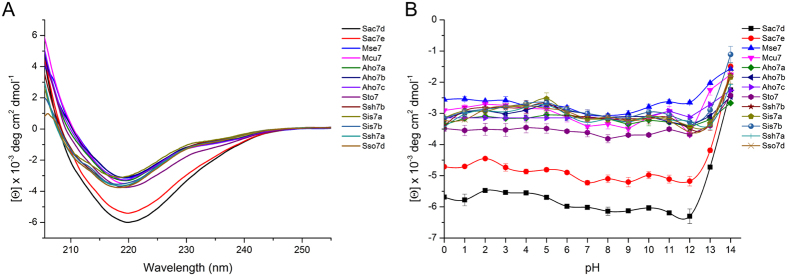
Characterization by circular dichroism. (**A**) CD spectra of proteins in 10 mM phosphate buffer, pH 7.4. (**B**) Study of the effect of pH on the protein structure. Proteins were incubated at room temperature overnight in a solution adjusted to each pH unit from pH 0 to pH 14 and the residual ellipticity were measured by CD at 222 nm. The continuous curves are drawn for clarity only.

To investigate whether the thirteen proteins had similar secondary structures, we used circular dichroism (CD) spectroscopy. These experiments were performed in a 10 mM phosphate buffer (pH 7.4) while omitting chloride salts to minimize noise for short wavelengths. Figure 4A shows that the shapes of CD spectra measured in the far-UV region were similar for the thirteen proteins. In correlation with the known crystallographic structures of Sac7d and Sso7d, these spectra were characteristic of proteins with an anti-parallel β-sheet and an α-helix^28^. As reported by Edmondson and Shriver^7^, we observed that Sac7d showed the lowest CD signal value at 222 nm^14^, likewise Sac7e, which is consistent with their longer α-helix region (Fig. 1A).

#### pH stability of the proteins

To study their pH stability, the proteins were incubated overnight at 20 °C in buffers at different pH from 0 to 14. CD measurements at 222 nm indicated that the secondary structure of all proteins remained largely stable under alkaline conditions up to pH 12 (Fig. 4B).

#### Thermostability of the proteins

The Supplementary Fig. S4 and Table 1 show that thermal stabilities of the proteins determined in PBS by differential scanning calorimetry (DSC) analysis ranged from 85.5 °C (Sac7e) up to 100 °C (Sto7). With an average temperature of 92.5 °C, these results show that all studied members from this family of proteins are hyperthermostable. DSC scans were characteristic of cooperative unfolding, indicating that variants were well folded. However, two behaviors were observed at high temperature. Mse7 and Mcu7 showed an irreversible unfolding, while all the other proteins showed no sign of aggregation after *T*_m_ was reached.

## Discussion

All the proteins produced were shown to be monomeric, with a native fold, and highly pH- and thermally-stable. They were able to bind various dsDNA sequences in PBS with affinities similar to those reported for Sac7d and Sso7d using similar buffers^18^,^19^ with a preference for sequences containing G/C bases. However, some interesting differences between proteins could be observed.

Despite the fact that all the sixteen residues known to be involved in DNA recognition^16^,^17^,^29^ are strictly conserved among the studied chromatin proteins, reflecting a strong selection pressure to maintain this function in the *Sulfolobales* order, variations could be observed for their dsDNA binding properties. For instance, according to fluorescence measurements, Ssh7b and Sac7e display the lowest and highest affinities of 22 ± 1 μM and 11 ± 1 μM, respectively. This is also seen with EMSA experiments for which Ssh7b is the only protein not showing a clear binding at 2.5 μM dsDNA, while Sac7e shows the strongest migration shift for 0.625 μM dsDNA. This could not be explained by an electrostatic effect, as we did not find a correlation between the affinities and the number of charges, which is quite homogeneous with a net charge of +7.8 ± 0.7 at pH 7.4 for all studied proteins. No correlation was found between helix and polypeptide chain lengths and affinities either. Ssh7b can be considered as a double mutant of Sis7b and Sso7d (V2T/E14Q and V2A/T17I, respectively, using Sac7d numbering), and a triple mutant of Sis7a and Ssh7a (V2T/E14Q/T17I and V2A/E14Q/T17I, respectively). The K_D_ values for Sis7a (15 ± 2 μM), Sis7b (16 ± 1 μM), Ssh7a (18 ± 2 μM) and Sso7d (17 ± 1 μM) are not significantly different, but are collectively significantly lower than that of Ssh7b (22 ± 1 μM). These observations suggest that the mutations E14Q and T17I alone are not responsible for the affinity variations, while their combination with position 2 seems to be important for affinity. However, other proteins with a valine in position 2, such as Sto7, display higher affinities. This highlights that variations of affinities in these small proteins are probably the result of slight readjustments in the structure induced by residue substitutions which are not being in direct contact with the ligand, the so called “second sphere” residues, as it was observed in other proteins such as antibodies^30^. Further mutagenesis and crystal structure studies will be needed to decipher the role of each position to fine-tune affinity. This study also shows that the three genes encoding 7 kDa DNA-binding proteins (Aho7a, Aho7b, and Aho7c) in *Acidianus hospitalis* are functional, as it was reported for the variants of *Sulfolobus acidocaldarius* and *Sulfolobus shibatae*^3^,^6^. These genes are probably resulting from duplication events, and further strengthen the idea that these chromatin proteins are important for the *Sulfolobales* order.

Our results show that all proteins are hyperthermostable in PBS 7.4, in good agreement with previous studies^31^. Two groups of proteins can be defined according to their thermostabilities: Sto7, Aho7c, Sso7d, Aho7b, Sis7a, Ssh7a, Aho7a (*T*m = 96.4 ± 1.7 °C; high *T*m group), and Sac7d, Ssh7b, Mcu7, Mse7, Sis7b, Sac7e (*T*m = 87.9 ± 1.5 °C; low *T*m group). No correlation can be found between the thermal stabilities of the studied proteins and the optimal growth temperatures of the host they come from (*Sulfolobus*, *Acidianus*, *Metallosphaera*). Interestingly, Sis7b (low *T*m group) can be considered as the single mutant I17T of Sis7a (high *T*m group) which has a deleterious effect on stability (−8.4 °C). This substitution at position 17 occurs in a partially buried region. In a previous study of the hydrophobic cores of Sac7d and Sso7d, Clark *et al.* hypothesized that this substitution could be partly responsible for the difference in stability between the two proteins^31^. Excluding Mse7 and Mcu7, which aggregated irreversibly upon heating, we found that all proteins from low *T*m group have a threonine at position 17. Our results confirm that an isoleucine is preferable to a threonine at this position in term of hydrophobicity and/or volume to contribute to the thermostability. The V30I substitution in Sac7d was shown by DSC to increase the thermal stability by 5.8 °C^31^. Interestingly, except Ssh7b and Sis7b, which both have the deleterious substitution I17T, all proteins from low *T*m group have a valine at position 30. None of the proteins from the high *T*m group have either the I17T or the V30I substitutions. This analysis shows and confirms that the positions 17 and 30 are two determinants for obtaining proteins with a higher thermostability. Thus, these positions, and probably their neighbouring ones, may represent interesting targets for mutagenesis studies aiming to stabilize proteins from this family, as this was done for other positions in Sso7d^32^. It is not obvious what other differences between proteins may be responsible for thermostability variations. Like for affinity, further studies are needed, for instance to understand what is responsible for the +3.2 °C increase observed between Aho7c and Sto7, the last being the most stable protein of this study (*T*m = 100 °C). It is also interesting to note that Mse7 and Mcu7, which are the most unrelated proteins to the others according to sequences, are presenting an aggregating behaviour. These two proteins are notably characterized by the solvent-exposed QDL sequence at positions 11 to 13, at the beginning of the second β-sheet, while every other variant presents an E(E/D)K sequence. Thus, Mse7 and Mcu7 display a smaller charge density in this area, trading three charged amino acids for only one. It is therefore likely that this more hydrophobic area could be involved in the aggregation of Mse7 and Mcu7 at high temperatures. Other substitutions not found in other proteins could also contribute to aggregation: V4I, I20V, R25K, as well as the particular sequence of their α-helix.

The proteins displayed a remarkable stability from pH 0 to at least pH 12. We previously reported that Sac7d and Sso7d are highly resistant from low to high pH^12^,^13^, and here, we confirm this as a general property of the “7 kDa DNA-binding” proteins. This is interesting considering that the pH inside *Sulfolobus* cells is about 6.55 while the pH outside is 3.5^33^, suggesting there has been no selection pressure for these pH resistances.

Finally, a comparison of published data^7^,^10^,^11^,^14^,^15^,^18^,^31^,^34^,^35^,^36^,^37^,^38^ with those reported in this work shows that the his-tag has no influence on the remarkable properties of Sac7d and Sso7d (monomeric state, affinities, CD spectra, stabilities), and most likely of the other members from this family. This is noteworthy for future studies of novel Sul7d members, as well as for the use of these proteins in applications requiring their detection.

Overall, this work shows that these proteins share similar high thermal/pH stabilities and DNA-recognition properties which are not so common to find in one small monomeric protein. This is why they are so attractive for various applications. In fact, we have not only one protein, but a natural repertoire provided by evolution. Indeed, we found differences which may be assets for some applications. Sso7d is principally used as a general DNA binding protein to develop molecular biology reagents with improved properties. Several studies have reported that processivity of different polymerases (Taq, Pfu, Tpa and KOD) can be greatly improved by fusion to Sso7d^39^,^40^,^41^,^42^. It might be interesting to use a more thermostable protein than Sso7d, Sto7 for example, to make reagents more resilient to PCR cycles at high temperature. For the design of artificial binders, a more stable scaffold is expected to withstand better high mutagenesis loads needed for their generation^43^. Also, a smaller size is interesting for *in vivo* applications, such as imaging and therapy, as it allows a better diffusion across barriers or to reach tumors for example^44^. Up to now, Sac7d and Sso7d have been used as scaffolds to design Affitins^13^,^21^,^24^,^25^. However, this study teaches us that Sto7 and Aho7c should be a good basis to generate more robust Affitins. We believe there will be no obvious reasons, but the historical ones, to continue using only Sac7d and Sso7d proteins for designing novel reagents.

As far as we know, this is the first time that a number of “7 kDa DNA-binding” proteins have been characterized simultaneously. With the continuous efforts to sequence whole genomes from archaeal microorganisms^1^, it is also likely that further discoveries will be made and the repertoire of this family of proteins will be extended.

## Materials and Methods

### Materials

Nucleic sequences corresponding to the thirteen homologous proteins studied in this work were computed by back translation of protein sequences registered in Uniprot database as P13123, P13125, A4YEA2, F4FYY6, F4B8×5, F4B9I5, F4B991, Q96×56, O59632, D2PHL8, F0NJT3, P61990, and P39476 using optimal codons for *E. coli*. These sequences were fully synthetized (GeneCust). Enzymes for molecular biology and DNA ladders were purchased from Thermo Fisher Scientific, calf thymus DNA from Sigma-Aldrich, Bugbuster from Novagen and oligonucleotides from Eurofins.

### Protein production

Nucleic sequences encoding the thirteen “7 kDa DNA-binding” proteins were cloned in pFP1001 expression vector^45^ between BamHI and HindIII restriction sites using T4 DNA ligase. The resulting ligations were used to transform the *E. coli* DH5α Iq cells that were spread on petri dishes. Fifty milliliters of 2 YT medium supplemented with 100 μg/mL ampicillin, 25 μg/mL kanamycin and 1% glucose were inoculated with a single colony and incubated overnight at 37 °C with shaking at 200 rpm. 20 mL of this culture were used to inoculate 1 L 2 YT medium supplemented with 100 μg/mL ampicillin, 25 μg/mL kanamycin and 0.1% glucose. After the optical density at 600 nm had reached a value of 0.8–1.0, expression of the cloned gene was induced by the addition of 0.5 mM Isopropyl β-D-1-thiogalactopyranoside (IPTG) and incubation at 30 °C for 3 h under shaking. Cells were pelleted by centrifugation (4500 × g) and the supernatants were discarded. Proteins were extracted with 15 mL TBS500 (20 mM Tris-HCl at pH 7.4, 500 mM NaCl) containing 25 mM imidazole, 25 mM MgSO_4_, 2 mg/mL lysozyme, 6 μg/mL DNAse I, and Bugbuster, Cell debris were pelleted by centrifugation for 10 min (8000 × g). Supernatants were purified by IMAC using a 1 mL column of Chelating Sepharose Fast Flow resin charged with Ni^2+^ (GE Healthcare) and equilibrated with TBS500 containing 25 mM imidazole. The resin was washed with 15 mL of this buffer, 20 ml of 20 mM Tris/2 M NaCl pH7,4 containing 25 mM imidazole and then with 20 mL of PBS (130 mM NaCl, 2.7 mM KCl, 10 mM Na_2_HPO_4_, 2 mM KH_2_PO_4_, pH 7.4) containing 25 mM imidazole. Purified proteins were eluted with PBS containing 250 mM imidazole. Proteins were then injected into a Superdex75 16/60 column (GE Healthcare) equilibrated with PBS and quantified spectrophotometrically at 280 nm using an extinction coefficient of 8250 M^−1^ cm^−1^. To check their monomeric state, purified proteins were loaded on an analytical Superdex 75 10/300 GL (GE Healthcare) column at a 500 μM concentration. The calibration proteins included: bovine serum albumin (BSA) (66 kDa), ovalbumin (44.3 kDa), ribonuclease A (13.7 kDa), and aprotinin (6.5 kDa). The Molecular weights of the studied proteins were checked by electrospray ionization liquid chromatography mass spectrometry (ESI-LC-MS).

### Detection of DNA-binding activity by Electrophoretic Mobility Shift Assay (EMSA)

For this assay, a 415 bp DNA fragment (42% G/C) was produced by polymerase chain reaction (PCR) amplification using pFP1001 containing the wild type *sac7d* gene as template, and Qe30for (5′-CTTTCGTCTTCACCTCGAG-3′) and Qe30rev (5′-GTTCTGAGGTCATTACTGG-3′) as primers. 200 ng of this dsDNA probe was incubated with different concentrations of the proteins (10 μМ, 5 μМ, 2.5 μМ, 1.25 μМ, 625 nМ) for 40 min at 25 °C. The total volume of each reaction was 10 μL in PBS. Samples were analyzed on 6% polyacrylamide gels in 45 mM Tris-borate buffer/1 mM EDTA at pH 8.0 (TBE). Gels were run at 80 V for 3 h, stained in TBE containing 1:10000 dilution of Gel-Red nucleic acid stain, scanned with GelDoc EZ Imager (Bio-Rad) and analyzed with Image Lab (Bio-Rad) software.

### Affinity measurements by fluorescence

ct-DNA was used for these experiments (42% G/C). 30 mg of lyophilized ct-DNA was dissolved in 0.2 M NaCl (10 mL) and incubated overnight at room temperature. ct-DNA was then fragmented on ice by sonication with a Vibracell sonicator for 3 min at 8 W. Obtained DNA fragments were evaluated on 1% agarose gel in TAE buffer (40 mM Tris, 20 mM acetic acid and 1 mM EDTA, pH 8.5). The ct-DNA sample was then dialyzed against PBS for 24 h at 4 °C and the ct-DNA concentration was determined spectrophotometrically at 260 nm using an extinction coefficient of 6600 M^−1^ cm^−1^, representing the concentration of the nucleotides in solution. Affinities between proteins and dsDNA from ct-DNA were measured by reverse titration with a spectrofluorometer FP-6500 (Jasco) as previously described^18^,^46^ with some modifications: excitation at 295 nm (3 nm band width), emission at 350 nm (5 nm band width) and protein concentration 5–6 μM. All measurements were performed at 25 °C in PBS.

Fluorescence titration data were analyzed using the following formula:


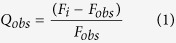


where F_i_ and F_obs_ represent the measured fluorescence in absence or presence of DNA, respectively. The fractional change in the fluorescence intensity due to quenching corresponds to the amount of DNA bound to a recombinant protein. It follows the equation:


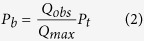


where P_b_ corresponds to the concentration of protein bound to DNA, P_t_ represents the total protein concentration in the solution, and Q_max_ is the maximum observed fluorescence quenching at higher concentrations of added DNA. The concentration of the free protein (P_f_) can be represented by the following equation:


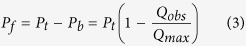


According to the model of McGhee - von Hippel^47^ for non-cooperative binding of proteins to DNA:


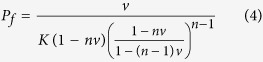


where K represents the binding constant, n corresponds to the size in nucleotides of the bound region of DNA by one protein molecule, and 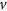 represents density of binding and can be defined by the equation:


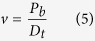


where D_t_ represents the final concentration of added DNA. As n, K and Q_max_ are unknown parameters, they were determined by means of a nonlinear least-squares fit of eq. 4 to the experimental data (Q_obs_) by an iterative procedure. For this purpose, an algorithm that iteratively solves equation (4) by bisection and then minimizes the weighted sum of squares of the deviations using the Levenberg–Marquardt algorithm was programmed in Visual Basic for Excel (Microsoft). The routine is available upon request.

### ELISA

ELISAs were performed using 96-well Maxisorb Nunc plates coated with 100 ng Neutravidin (Pierce) in PBS mainly as previously described^48^. To produce the 40 bp dsDNA, targets the 3′-biotinylated plus-strand oligonucleotides poly(dAdT)_20_, poly(dAdC)_20_, poly(dAdG)_20_, poly(dGdC)_20_, and poly(dA)_40_, were annealed with their complementary ones at the ratio of 2:3 by incubation for 30 s at 90 °C followed by slow cooling to 20 °C. Then, 2.5 ng of these biotinylated dsDNA in PBS was immobilized in each well. 100 μL of 200 nM purified proteins in 10 mM KH_2_PO_4_ 50 mM KCl, pH 7 containing 0.1% Tween 20, were used to test the binding to dsDNA. Binding to BSA was used as a negative control. The detection was performed as described previously using the RGS His6 antibody horseradish peroxidase (HRP) conjugate (Qiagen) which detects the RGS His6-tag from proteins^12^.

### Circular dichroism measurements

Circular dichroism (CD) spectra and the monitoring of ellipticity *vs* pH were done for each protein as published elsewhere for Affitins^12^,^13^.

### Thermostability Measurements

DSC experiments were carried out in PBS as described previously^49^ using a VP-DSC instrument (Microcal, Northampton, MA) and data was analyzed with the software supplied with the equipment.

## Additional Information

**How to cite this article**: Kalichuk, V. *et al.* The archaeal “7 kDa DNA-binding” proteins: extended characterization of an old gifted family. *Sci. Rep.* **6**, 37274; doi: 10.1038/srep37274 (2016).

**Publisher’s note:** Springer Nature remains neutral with regard to jurisdictional claims in published maps and institutional affiliations.

## Supplementary Material

Supplementary Information

## Figures and Tables

**Table 1:** Properties of the recombinant proteins.

Protein	M.W. (Da.)^a^	Yield (mg/L)^b^	*K*_*D*_ (μM)^c^	n (bases)^d^	*T*m (°C)^e^
Sac7d	9108 (9103)	8.0	13 ± 1	6, 4 ± 0, 2	89, 6
Sac7e	8968 (8964)	5.5	11 ± 1	6, 9 ± 0, 4	85, 5
Mse7	8477 (8473)	6.5	15 ± 1	6, 2 ± 0, 3	87, 4^*^
Mcu7	8449 (8446)	7.0	16 ± 1	6, 7 ± 0, 2	88, 8^*^
Aho7a	8533 (8531)	5.0	16 ± 1	6, 1 ± 0, 1	94, 7
Aho7b	8618 (8615)	6.0	13 ± 1	5, 7 ± 0, 3	95, 8
Aho7c	8374 (8372)	8.0	16 ± 1	5, 7 ± 0, 2	96, 8
Sto7	8808 (8804)	8.5	14 ± 1	6, 5 ± 0, 3	100, 0
Ssh7b	8795 (8790)	10.0	22 ± 1	6, 3 ± 0, 2	89, 0
Sis7a	8806 (8803)	8.0	15 ± 2	6, 7 ± 0, 4	95, 6
Sis7b	8797 (8791)	7.0	16 ± 1	6, 0 ± 0, 3	87, 2
Ssh7a	8776 (8773)	7.0	18 ± 2	7, 9 ± 0, 4	95, 6
Sso7d	8778 (8774)	8.0	17 ± 1	8, 1 ± 0, 3	96, 5

^a^Molecular weights determined by mass spectrometry; those calculated from sequences including the tag (see legend of Fig. 1A) are in brackets.

^b^mg of purified protein obtained from 1 L growth medium.

^c,d^Dissociation constants and site sizes of DNA binding from fluorescence experiments.

^e^Melting temperatures from DSC experiments,

^*^aggregation at high temperatures.
