# Supplementary material for: The archaeal “7 kDa DNA-binding” proteins: extended characterization of an old gifted family
Source: Sci Rep. 2016 Nov 17;6:37274. doi: 10.1038/srep37274 (PMC5112516; doi:10.1038/srep37274)
Supplement: Supplementary Information [file srep37274-s1.pdf]

Supplementary information

The archaeal "7 kDa DNA-binding" proteins: extended characterization of an old gifted family

Valentina Kalichuk[1,2,†], Ghislaine Béhar[1,†], Axelle Renodon-Cornière[1], Georgi Danovski[1], Gonzalo Obal[3], Jacques Barbet[1], Barbara Mouratou[1,*], Frédéric Pecorari[1,*]

1 CRCNA, Inserm, CNRS, Université d'Angers, Université de Nantes, Nantes, France,

2 Université catholique de Louvain, Louvain Drug Research Institute, Advanced Drug Delivery and Biomaterials, Brussels, Belgium,

3 Institut Pasteur de Montevideo, Protein Biophysics Unit, Montevideo, Uruguay.

[†] These authors contributed equally to the paper as first authors.

[*] To whom correspondence should be addressed.
Dr. Frédéric Pecorari, Tel.: +33 2 40 41 28 51; Fax: +33 2 28 08 02 04; E-mail: frederic.pecorari@univ-nantes.fr,
Dr. Barbara Mouratou, Tel.: +33-2 40 41 28 51, Fax: +33 2 28 08 02 04; E-mail: barbara.mouratou@univ-nantes.fr.

SUPPLEMENTARY FIGURES

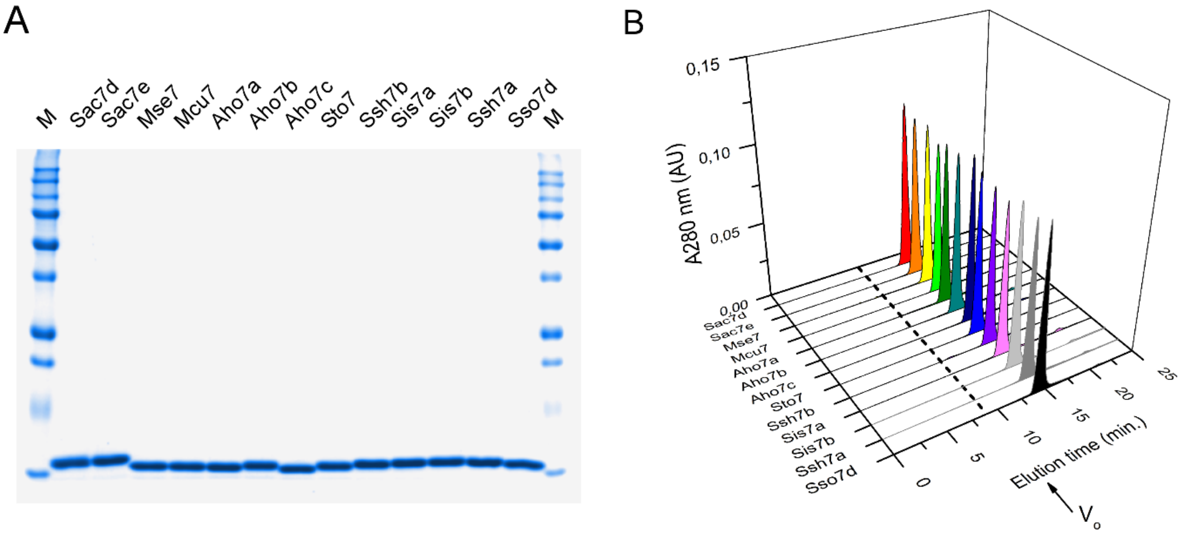

Figure S1. Characterization of the molecular weights of the proteins. (A) SDS-PAGE analysis of the purified proteins. The proteins (1 µg) were analysed on 15% polyacrylamide gel after IMAC and gel filtration purification. Lane M corresponds to protein markers: 250, 150, 100, 75, 50, 37, 25, 20, 15, 10 kDa from top to bottom. (B) Gel filtration analysis of the proteins. All proteins were loaded on an analytical Superdex 75 10/300 GL (Vt = 24 mL) at a concentration of 500 µM. Vo indicates the void volume of the column determined by injection of dextran.

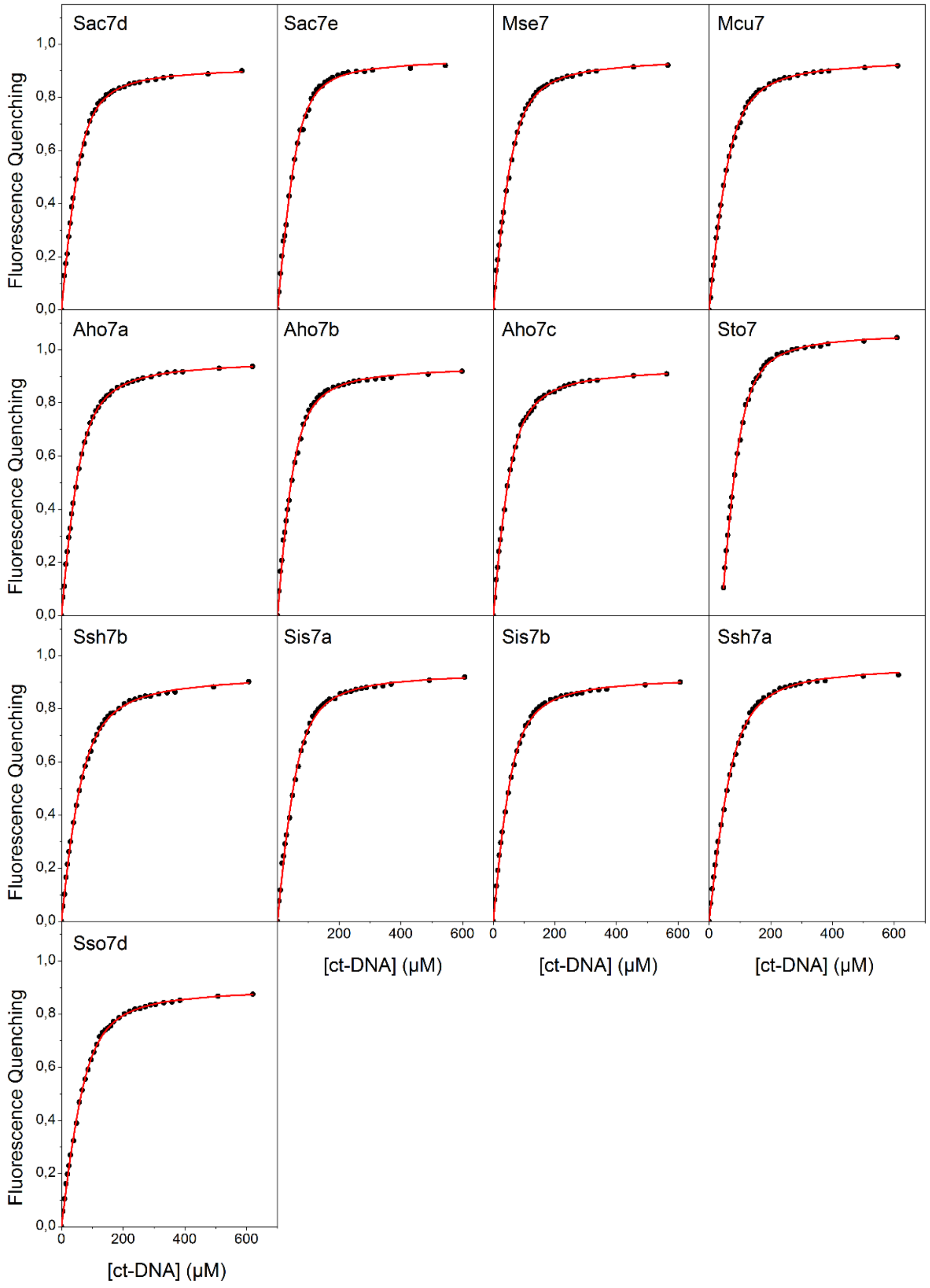

Figure S2. Affinities between proteins and dsDNA. Affinity measurements were performed by reverse titration of proteins with ct-DNA in PBS pH 7.4 at 25°C monitored by quenching of intrinsic tryptophan fluorescence intensity at 350 nm. Data (•) were analysed using the McGhee-von Hippel model (red curve) to determine K_D.

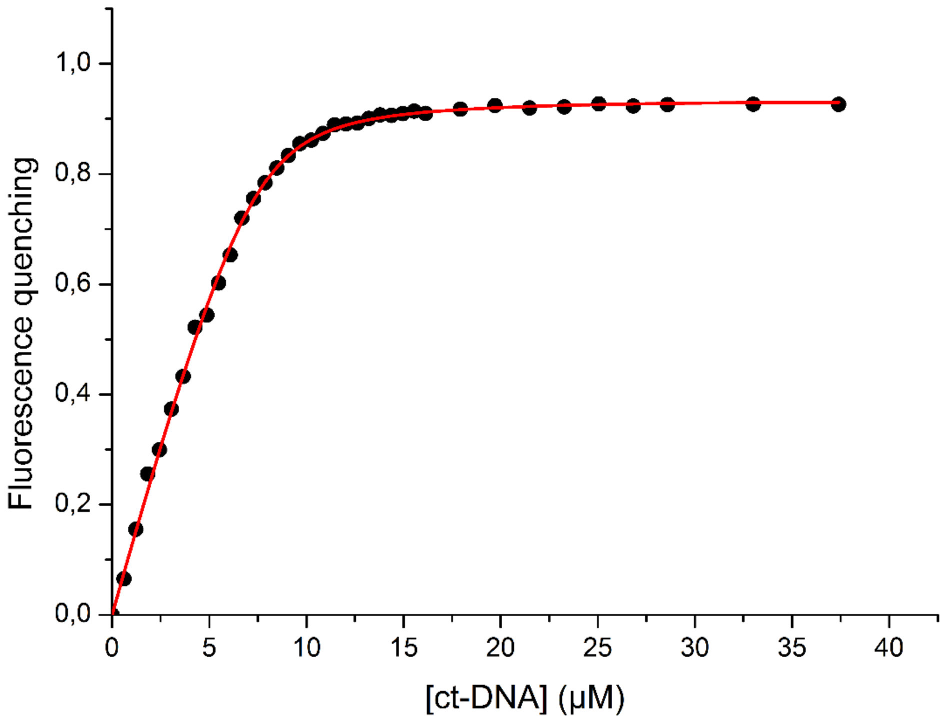

Figure S3. Affinity between Sac7d and dsDNA. Affinity measurement was performed by reverse titration of proteins with ct-DNA in 10 mM KH_2PO_4, 50 mM KCl, pH 7 at 25°C monitored by quenching of intrinsic tryptophan fluorescence intensity at 350 nm. Data (•) were analyzed with McGhee-von Hippel model (red curve) to determine K_D.

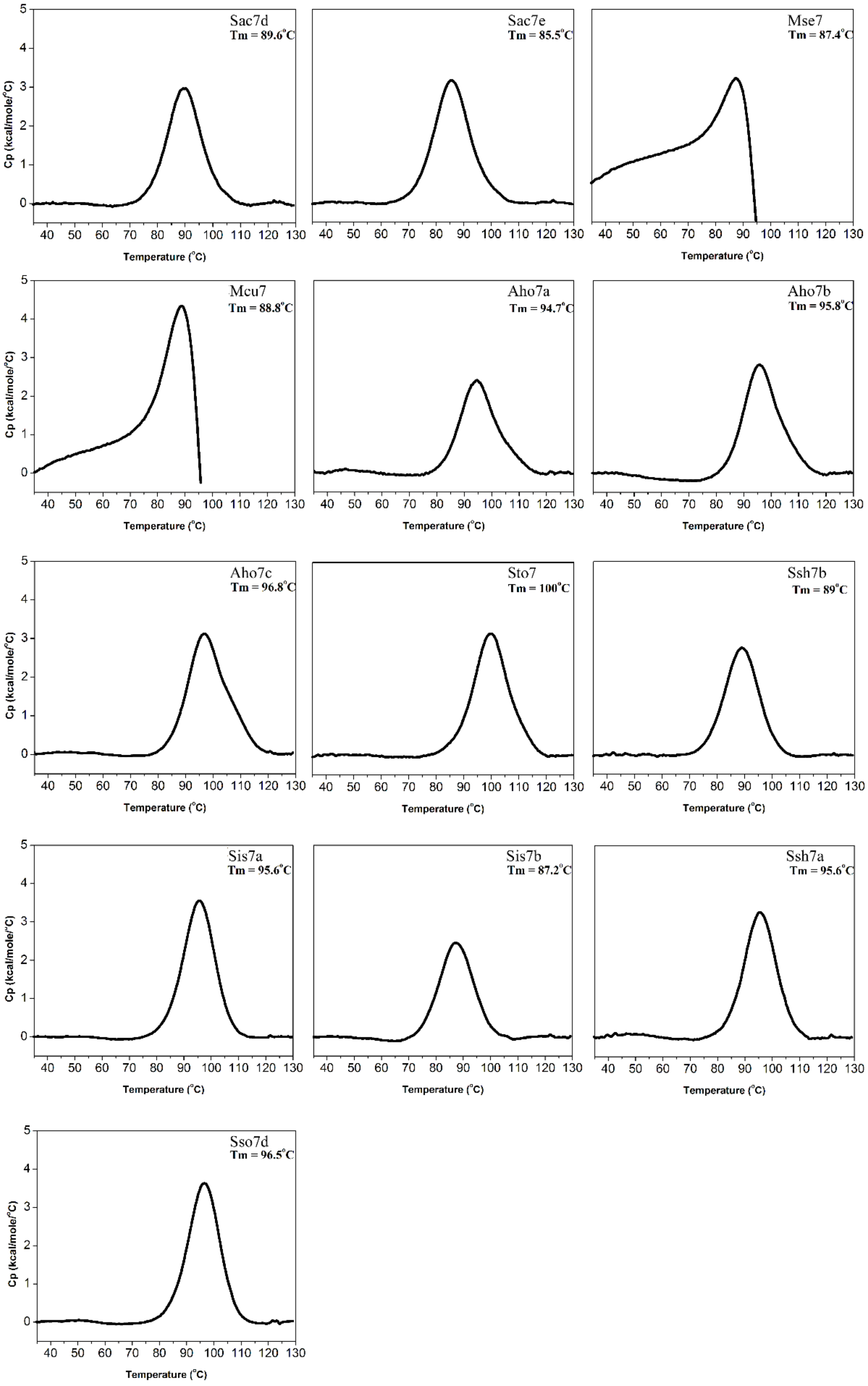

Figure S4. Thermal stabilities of proteins. DSC curves were recorded for proteins in PBS 7.4.
